# Intensive longitudinal assessment of mobility, social activity and loneliness in individuals with severe mental illness during COVID-19

**DOI:** 10.1038/s41537-023-00383-8

**Published:** 2023-09-20

**Authors:** Linda Valeri, Habiballah Rahimi-Eichi, Einat Liebenthal, Scott L. Rauch, Russell K. Schutt, Dost Öngür, Lisa B. Dixon, Jukka-Pekka Onnela, Justin T. Baker

**Affiliations:** 1 Department of Biostatistics, Columbia University, New York, NY USA; 2 Harvard Medical School, Boston, MA USA; 3 McLean Hospital, Belmont, MA USA; 4 University Of Massachusetts, Boston, MA USA; 5 Beth Israel Deaconess Medical Center, Boston, MA USA; 6 Columbia University, New York, NY USA; 7 New York State Psychiatric Institute, New York, NY USA; 8 Harvard T.H. Chan School of Public Health, Boston, MA USA

**Keywords:** Schizophrenia, Human behaviour

## Introduction

Social isolation and loneliness worsen symptoms and functioning among individuals diagnosed with severe mental illness (SMI)^1,2^. However, there is a dearth of studies on changes in social activity due to COVID-19 shelter-in-place-orders and how they might impact perception of loneliness. Additionally, to our knowledge, no data have been published on changes in mobility patterns during the pandemic, specifically regarding people with SMI. Mobility metrics can be derived from passively collected smartphone Global Positioning System (GPS) receivers and are important correlates of physical, cognitive, and mental health in chronic illness. Recent evidence suggests the potential of measuring mobility in the SMI population, given that increased time spent at home has been linked to neurocognitive function^3^, negative symptoms and community functioning in people with schizophrenia^4^, and depressive states in patients with bipolar disorder^4^. For adults with SMI, home can be a context of loneliness and isolation. Shelter-in-place orders, by limiting mobility, can influence the potential of social interactions outside home, thereby impacting loneliness perceptions. However, the potential influence of shelter-in-place orders, intended specifically to restrict mobility, on social interactions and subsequent loneliness perceptions remains unknown.

While studies among the general population have documented a shift from in-person to digital social activity during the COVID-19 lockdowns^5,6^, findings regarding the effect of this shift on feelings of loneliness have been inconsistent. Some studies have reported increased levels of loneliness during COVID-19 lockdowns^7^, while others have found relatively stable levels of perceived loneliness under such strict measures^8–10^.

There is a significant knowledge gap in understanding how the pandemic has affected social behavior, mobility, and loneliness perception in individuals with bipolar disorder and schizophrenia. Furthermore, it is unclear whether changes in social activity and/or mobility may mediate the impact of the lockdown on loneliness. Only one study reported a perception of reduced social support and no changes in loneliness when comparing the COVID-19 lockdown and pre-lockdown periods^11^. Notably, while this study collected longitudinal information, there was only one time point prior and six time points post COVID-19 shelter-in-place orders.

Given their established link with severity and progression of SMI^12,13^, it is critical to assess the impact of the COVID-19 pandemic on behavioral traits, such as social activity and mobility, for people with SMI along with describing changes in loneliness perceptions.

Moreover, understanding the effects of shelter-in-place orders on these factors could help explain the mixed evidence regarding the impact of the pandemic on mental health outcomes in bipolar disorder and schizophrenia^14^. Recent research suggests that individuals with bipolar disorder experienced low rates of relapse and symptom exacerbation^15,16^, less severe psychiatric symptoms^17^, and higher levels of well-being^18^ during the pandemic compared to the pre-pandemic period. Conversely, studies involving individuals with schizophrenia have reported symptom exacerbation during the pandemic^19^. These differing findings underscore the need for further research on how the pandemic has affected, potentially in a different fashion, the living conditions, behaviors, and ultimately the mental health of individuals with SMI.

**Fig. 1:**
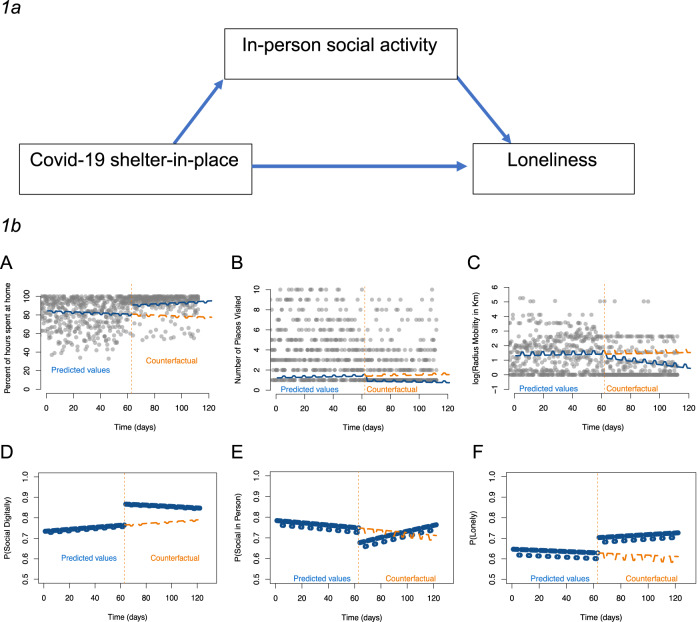
Graphical representation of the hypothesis evaluated in our study and results for interrupted time series analyses. **1a** Graphical representation of the hypothesis evaluated in our study. We hypothesize that the effect of Covid-19 shelter-in-place orders on loneliness are mediated by a reduction of in-person social activity during the pandemic compared to levels of in-person social activity prior the shelter-in-place orders. **1b** Results for interrupted time series analyses. Effect of shelter-in-place orders on (A) daily percent of time spent at home (B) daily number of points of interest visited (C) daily log of radius mobility (D) probability to be a little to extremely active in digital social activity (E) probability to be a little to extremely active in in-person social activity and (F) probability to be a little to extremely lonely. Blue lines indicate predicted outcomes before and after the shelter in place order from our models. Orange lines indicate predicted outcomes had shelter in place orders not been enacted (counterfactual).

The adoption of smartphone technology to monitor behaviors and symptoms in the SMI population continuously over time has the potential to enhance measurement precision and statistical power to detect the effects of the pandemic on social activity, mobility, and loneliness^20^. However, we are not aware of longitudinal smartphone studies in the SMI population that were initiated prior to the pandemic and continued to monitor the subjects during shelter-in-place orders. We therefore conducted an intensive longitudinal pilot study to evaluate the impact of shelter-in-place orders on social activity, mobility, and loneliness among individuals diagnosed with a bipolar disorder or schizophrenia. Our study focused on a subset of participants from the Intensive Longitudinal Health Behavior Network who had active smartphone data collection for at least two months before and after the implementation of shelter-in-place orders. In this pilot study, we aimed to investigate the mediating role of social activity and mobility patterns in explaining the relationship between COVID-19 shelter-in-place orders and loneliness. Social activity was assessed through self-reported daily digital social activity and in-person social activity using an ordinal scale as well as loneliness perceptions. Mobility patterns were constructed from smartphone GPS data. We proposed two hypotheses. First, our hypothesis suggests that the impact of COVID-19 shelter-in-place orders on loneliness is partly influenced by a reduction in social interactions during the pandemic compared to pre-pandemic levels^21,22^. We believe that this decrease in social interactions can lead to a greater sense of disconnection from one’s social networks, consequently increasing feelings of loneliness^23^. Secondly, we hypothesize that the mediating effect will be more significant for in-person social activities compared to digital social activities. This hypothesis is based on studies conducted with individuals living with psychosis, which have shown that substituting in-person interactions with digital communication may intensify loneliness and exacerbate existing mental symptoms^24–26^. We anticipate that any potential increase in digital social activity during the pandemic would not be sufficient to counterbalance the effects of shelter-in-place orders on loneliness in this population. Figure 1a illustrates our hypotheses. To the best of our knowledge, this is the first study to investigate these hypotheses among individuals with SMI using daily information on participants’ mobility and daily surveys conducted before and during the pandemic.

## Methods

A total of 74 individuals diagnosed with SMI were enrolled in the study between 2015 and 2021 and followed for up to 4 years as part of the Intensive Longitudinal Health Behavior Network project, which utilizes harmonized methods across multiple disease areas. Adults with Psychosis (Schizophrenia, Bipolar Disorder with Psychotic Features, Schizoaffective Disorder) were eligible to participate in the study. Participants provided written informed consent, and the study was approved by the local Institutional Review Board.

For data collection, participants completed daily surveys on digital and in-person social activity as well as loneliness using the Beiwe smartphone application^27^. Loneliness was assessed using a categorical item with four response options ranging from ‘not at all’ to ‘extremely’.

In this analysis, we adopted a granular approach and analyzed data from participants who had been followed for at least two months prior to the enactment of shelter-in-place orders on March 24, 2020, in Massachusetts, and for at least two months thereafter. Nine participants met these criteria.

Among the nine participants involved in the study, data from seven participants were of high quality and utilized for deriving daily mobility measures. This was made possible through the utilization of an openly available pipeline, DPLocate, developed by the authors^28,29^. DPLocate is a software that securely processes sparsely collected raw GPS data from smartphone devices, extracting behavioral patterns related to returning to points of interest (POIs) within specific time intervals^29^. A POI refers to a location where the user usually spends a significant amount of time^30^.

In addition to generating daily maps illustrating relocation patterns as reported in ref. ^31^, the pipeline also computed various metrics previously defined in relevant publications^29^. These metrics encompassed the percentage of staying at home during the day in percent, the number of POIs other than home visited during the day, and the radius of the circle that encompasses all visited locations during the day in Kilometers (see supplementary materials for details).

Our primary predictor variable was the COVID-19 shelter-in-place order, which was operationalized to reflect whether the daily measures of mobility and self-reported social behavior and loneliness were collected before or after March 24, 2020, the date when the shelter-in-place orders were enacted in Massachusetts, where all the participants resided. These orders included restrictions on gatherings of more than ten people and the closure of non-essential businesses, which lasted from March 24th, 2020, to April 7th, 2020^32^.

To quantify the effect of shelter-in-place orders on digital and in-person social activity, daily mobility measures, and loneliness, we utilized generalized linear mixed models, adjusting for temporal trends. In addition, longitudinal mediation analyses^33^ were conducted to examine the role of changes in social activity following the shelter-in-place orders to explain the effect of these orders on the trajectory of loneliness. Denoting shelter-in-place order as the exposure, loneliness as the outcome, digital and in-person social activity and mobility measures as potential mediators, we then proceeded evaluating evidence of mediation. The direct effect of shelter-in-place orders on loneliness and the indirect effect mediated through changes in social activity and mobility were estimated if a mediator-outcome association was successfully detected, and statistical significance was assessed using a two-sided *p*-value (<0.05). In a sensitivity analysis, we further adjusted for diagnosis (primary psychotic or primary affective disorder). All analyses were performed using Rstudio version 1.3.1073^34^. Methodological details can be found in the supplementary materials (eMethods). This study adhered to the Strengthening the Reporting of Observational Studies in Epidemiology (STROBE) reporting guideline^35^.

## Results

Table 1 presents the descriptive statistics for our sample of seven participants, for whom we collected 634 complete daily observations of survey and passive data over a four-month period, including two months before and two months after the shelter-in-place orders were implemented. Three males and four females were included in our sample and the mean age was 29.

**Table 1:** Descriptive Statistics on demographics and for survey items and mobility features two months before and after shelter-in-place orders for *n* = 7 subjects.

	2 months before Shelter-in-place order		2 months after Shelter-in-place order	
	*Mean/count*	*SD/%*	*Mean/count*	*SD/%*
**Demographics**				
Age	29	9.6	29	9.6
Male	3	43%	3	43%
Bipolar	5	71%	5	71%
**Survey data**				
**Loneliness**				
	1.48	1.17	1.51	1.09
Not at all	104	23%	66	16%
A little	62	14%	75	18%
Moderately	90	20%	73	17%
Extremely	92	20%	69	16%
NA	93	21%	130	31%
**Social Digitally**				
	1.58	0.95	1.70	0.72
Not at all	60	13%	11	2%
A little	82	18%	97	23%
Moderately	150	34%	144	35%
Extremely	56	12%	34	8%
NA	93	21%	127	30%
**Social in person**				
	1.62	0.97	1.32	1.00
Not at all	61	13%	80	19%
A little	72	16%	63	15%
Moderately	153	34%	110	26%
Extremely	63	14%	32	7%
NA	92	20%	128	30%
**Mobility Features**				
**Daily Percent time spent at home**	82.5	16	97	10
**Daily Radius travelled (Km)**	9.2	21.8	3.9	15.5
**Daily Number of POIs**	4.1	3.1	2.3	2.1

Our analyses revealed that the COVID-19 shelter-in-place orders had a significant impact on participants’ social activity and mobility patterns. Within one month of the orders being enacted, there was a reduction in self-reported in-person social activity (ORpre/post = 0.67, *p*-value = 0.02) and an increase in digital social activity (ORpre/post = 4.21, *p*-value < 0.001). Participants also reported spending a higher percentage of their daily hours at home (RDpre/post = 13.79, *p*-value < 0.001), traveling shorter distances (RDpre/post = −0.59, *p*-value = 0.02), and visiting fewer points of interest (RRpre/post = 0.44, *p*-value < 0.001) after the shelter-in-place orders (Fig. 1b).

Furthermore, the shelter-in-place orders were associated with an increase in loneliness, although it did not reach statistical significance (ORpre/post = 2.10, *p*-value = 0.08). Mediation analysis suggested that the reduction in in-person social activity partially mediated the effect of the shelter-in-place orders on the probability of feeling lonely (direct effect (DE) on the difference scale: DE = 0.05, *p*-value = 0.04; indirect effect (IE): IE = 0.021, *p*-value < 0.001; proportion mediated: 30%). The indirect effect quantifies how much the changes in in-person social activity following shelter-in-place orders mediated changes in loneliness. The direct effect is the effect of shelter-in-place orders on loneliness that operates independently of in-person social activity. These results indicate that changes in participants’ in-person social activity patterns played a role in their experience of loneliness. However, digital social activity and daily mobility patterns did not show a significant association with loneliness in this sample (refer to the supplementary materials for detailed regression and mediation analysis results).

Overall, these findings provide insights into the impact of the COVID-19 shelter-in-place orders on social activity, mobility, and loneliness among our sample of individuals with SMI.

## Discussion

This study presents pilot data demonstrating the effectiveness of using smartphones to monitor loneliness, social activity, and mobility among individuals with SMI. This approach enhances our ability to capture complex perceptions and behaviors in situ, especially when monitoring individuals in naturalistic settings. Although the small sample size limits the generalizability of our findings and unmeasured confounding might introduce bias in the mediation analyses, our results provide insights into the effects of the COVID-19 pandemic on social activity, mobility, and loneliness.

Despite the limitations, our study indicates that changes in in-person social activity following shelter-in-place orders may partially mediate the effect of shelter-in-place orders on changes in loneliness experienced during the pandemic. Specifically, the decrease in in-person social activity appears to contribute to the increased feelings of loneliness compared to the pre-pandemic period. Conversely, we did not find evidence of mediation effects for mobility and digital social interaction during the pandemic.

The observed strong effects of shelter-in-place orders on mobility and social activity measures, along with suggestive evidence of an association with increased loneliness, highlight the value of adopting digital technologies to gain a deeper understanding of the connections between behavior and symptoms in a more nuanced manner. These approaches provide opportunities for identifying behavioral targets for treatments and improving clinical decision-making through close monitoring of patients in clinical settings. Additional research will be needed to determine the extent to which these changes in loneliness may have impacted symptoms and functioning of those with serious mental illness.

While further research is needed to validate and expand upon these preliminary findings, our study highlights the potential benefits of leveraging digital technologies and wearable devices for enhanced monitoring and improved intervention strategies for individuals with SMI.

### Reporting summary

Further information on research design is available in the Nature Research Reporting Summary linked to this article.

### Supplementary information

Supplementary Tables and Figures

Appendix

reporting summary

## Data Availability

R code of the analyses presented in this paper is available upon request from the corresponding author.
