# Supplementary material for: Intensive longitudinal assessment of mobility, social activity and loneliness in individuals with severe mental illness during COVID-19
Source: Schizophrenia (Heidelb). 2023 Sep 20;9(1):62. doi: 10.1038/s41537-023-00383-8 (PMC10511540; doi:10.1038/s41537-023-00383-8)
Supplement: Supplementary file 1 — Supplementary Tables and Figures [file 41537_2023_383_MOESM1_ESM.pdf]

Intensive Longitudinal Assessment of Mobility, Social Activity and Loneliness in Individuals with Severe Mental Illness During COVID-19

Authors: Linda Valeri, Habiballah Rahimi-Eichi, Einat Liebenthal, Scott L. Rauch, Russell Schutt, Dost Ongur, Lisa Dixon, *Jukka-Pekka Onnela, *Justin Baker

*Co-last authors

Supplementary Tables

Table 1S Descriptive Statistics for survey variables 2 months before and after shelter-in-place orders for 9 participants

	**2 months before Shelter-in-place order**		**2 months after Shelter-in-place order**	
	Count	*%*	*Count*	*%*
**Loneliness**				
Not at all	134	24%	116	20%
A little	92	16%	103	18%
Moderately	102	18%	100	18%
Extremely	89	16%	73	13%
NA	132	24%	166	30%
**Social Digitally**				
Not at all	84	15%	54	10%
A little	100	18%	113	20%
Moderately	178	32%	197	35%
Extremely	54	10%	34	6%
NA	133	24%	160	28
**Social in person**				
Not at all	88	16%	120	21%
A little	83	15%	72	13%
Moderately	187	34%	172	30%
Extremely	60	13%	33	6%
NA	131	23	161	28%

Table 2S Descriptive Statistics for survey variables 6 months before and after shelter-in-place orders for 9 participants

	**6 months before Shelter-in-place order**		**6 months after Shelter-in-place order**	
	Mean/count	*SD/%*	*Mean/count*	*SD/%*
**Loneliness**				
Not at all	308	19%	272	16%
A little	260	16%	201	12%
Moderately	229	14%	172	10%
Extremely	145	9%	116	7%
NA	705	42%	895	54%
**Social Digitally**				
Not at all	194	12%	135	8%
A little	244	15%	232	14%
Moderately	426	26%	352	21%
Extremely	78	5%	46	3%
NA	705	42%	891	54%
**Social in person**				
Not at all	151	9%	209	8%
A little	183	11%	180	14%
Moderately	483	30%	317	21%
Extremely	127	8%	60	3%
NA	703	42%	890	54%

Table 3S Generalized linear mixed model analyses with survey answers recorded 2 months pre and post shelter-in-place orders in Massachussetts

	loneliness			social digitally			social in person		
Predictors	*Odds Ratios*	*CI*	*p*	*Odds Ratios*	*CI*	*p*	*Odds Ratios*	*CI*	*p*
(Intercept)	1.95	0.28 – 13.61	0.502	83.27	0.01 – 1323538.55	0.370	7.09	0.68 – 74.11	0.102
shelter	2.10	0.90 – 4.91	0.086	14.55	3.58 – 59.12	**<0.001**	0.89	0.39 – 2.02	0.780
day_c	1.00	0.98 – 1.01	0.733	0.99	0.97 – 1.01	0.429	0.99	0.97 – 1.00	0.144
weekday	1.28	0.78 – 2.09	0.323	1.32	0.63 – 2.77	0.467	1.96	1.18 – 3.27	**0.010**
shelter * day_c	1.01	0.98 – 1.03	0.575	1.00	0.96 – 1.04	0.850	1.01	0.99 – 1.04	0.317

Table 4S Generalized linear mixed model analyses with GPS data and survey answers recorded 2 months pre and post shelter-in-place orders in Massachussetts

	percent Home			Number of Places			Log(Radius Mobility in Km)		
Predictors	*Estimates*	*CI*	*p*	*Incidence Rate Ratios*	*CI*	*p*	*Estimates*	*CI*	*p*
(Intercept)	79.07	72.09 – 86.06	**<0.001**	4.75	3.50 – 6.46	**<0.001**	1.66	1.20 – 2.13	**<0.001**
shelter	9.89	6.38 – 13.39	**<0.001**	0.60	0.50 – 0.71	**<0.001**	-0.29	-0.64 – 0.06	0.099
day_c	-0.06	-0.12 – 0.01	0.100	1.00	1.00 – 1.01	0.078	0.00	-0.00 – 0.01	0.591
weekday	1.81	-0.35 – 3.97	0.101	0.85	0.76 – 0.94	**0.002**	-0.24	-0.46 – -0.03	**0.028**
shelter * day_c	0.13	0.03 – 0.24	**0.013**	0.99	0.99 – 1.00	0.074	-0.01	-0.02 – -0.00	**0.010**

Table 5S Generalized linear mixed model for the association of social activity on loneliness recorded up to 2 months pre and post shelter-in-place orders in Massachussetts

	Loneliness Adj SIP & SD		
Predictors	*Odds Ratios*	*CI*	*p*
(Intercept)	15.03	1.19 – 190.33	**0.036**
shelter	2.54	1.05 – 6.17	**0.039**
day_c	1.00	0.98 – 1.01	0.557
weekday	1.38	0.83 – 2.30	0.211
social_digitally	0.46	0.13 – 1.65	0.235
social_in_person	0.15	0.06 – 0.34	**<0.001**
shelter * day_c	1.00	0.98 – 1.03	0.833
shelter * social_digitally			
shelter * social_in_person			

Table 6S Generalized linear mixed model for the association of mobility patterns with loneliness recorded up to 2 months pre and post shelter-in-place orders in Massachussetts

	Loneliness Adj %home			Loneliness Adj Num places			Loneliness Adj Radius		
Predictors	*Odds Ratios*	*CI*	*p*	*Odds Ratios*	*CI*	*p*	*Odds Ratios*	*CI*	*p*
(Intercept)	0.86	0.06 – 11.78	0.911	4.47	0.58 – 34.67	0.152	3.33	0.44 – 25.14	0.244
shelter	1.85	0.67 – 5.08	0.232	1.93	0.71 – 5.22	0.197	2.17	0.81 – 5.78	0.121
day_c	1.00	0.98 – 1.01	0.717	1.00	0.98 – 1.02	0.760	1.00	0.98 – 1.01	0.680
weekday	1.32	0.75 – 2.34	0.339	1.30	0.73 – 2.31	0.372	1.38	0.78 – 2.43	0.271
percentHome	1.02	0.99 – 1.04	0.164						
shelter * day_c	1.01	0.98 – 1.04	0.368	1.01	0.98 – 1.04	0.398	1.01	0.98 – 1.04	0.366
numPlaces				0.93	0.84 – 1.03	0.150			
radiusMobility_log							0.93	0.73 – 1.19	0.584

Table 7S Generalized linear mixed model analyses with survey answers recorded up to 6 months pre and post shelter-in-place orders in Massachussetts

	loneliness			social digitally			social in person		
Predictors	*Odds Ratios*	*CI*	*p*	*Odds Ratios*	*CI*	*p*	*Odds Ratios*	*CI*	*p*
(Intercept)	2.68	0.53 – 13.65	0.235	23.30	0.92 – 590.13	0.056	14.02	1.14 – 172.55	**0.039**
shelter	1.36	0.80 – 2.33	0.256	7.72	3.38 – 17.66	**<0.001**	0.50	0.28 – 0.88	**0.016**
day_c	1.01	1.00 – 1.01	**<0.001**	1.01	1.00 – 1.01	**0.007**	0.99	0.99 – 1.00	**0.005**
weekday	1.34	0.99 – 1.81	0.059	1.09	0.72 – 1.66	0.675	1.26	0.89 – 1.80	0.194
shelter * day_c	0.98	0.98 – 0.99	**<0.001**	0.98	0.97 – 0.99	**0.001**	1.02	1.01 – 1.03	**<0.001**

Table 8S Generalized linear mixed model for the effect of social activity on loneliness (sample including 6 months pre and post shelter-in-place orders in Massachussetts)

	Loneliness Adj SIP & SD		
Predictors	*Odds Ratios*	*CI*	*p*
(Intercept)	20.05	2.48 – 162.25	**0.005**
shelter	1.32	0.76 – 2.30	0.324
day_c	1.01	1.00 – 1.01	**<0.001**
weekday	1.35	0.99 – 1.84	0.056
social_digitally	0.56	0.28 – 1.15	0.116
social_in_person	0.14	0.07 – 0.26	**<0.001**
shelter * day_c	0.99	0.98 – 0.99	**<0.001**
shelter * social_digitally			
shelter * social_in_person			

Table 9S Mediation analysis of the role of social activity in person as mediator of the COVID-19 emergency declaration effect on loneliness. Estimates on the difference in probability scale (2 and 6 months pre-post shelter-in-place orders). Outcome and mediator models adjusted for temporal confounding (weekday and day). In sensitivity analyses further adjusting for patients' diagnosis conclusions of the analyses did not change.

	2 months pre/post	2 months pre/post + dx	6 months pre/post	6 months pre/post + dx
	Social activity in person	Social activity in person	Social activity in person	Social activity in person
Direct effect	0.088 (-0.019,0.19)	0.075 (-0.015,0.19)	0.049 (-0.001,0.11)	0.052 (-0.008,0.14)
Indirect effect	0.003 (-0.01,0.03)	0.001 (-0.019,0.02)	0.02 (0.003,0.04)	0.021 (0.002,0.04)
Total effect	0.090 (-0.016,0.19)	0.077 (-0.019,0.20)	0.070 (0.01,0.14)	0.074 (0.01,0.18)
Proportion mediated	3%	3%	28%	30%

Supplementary Figures

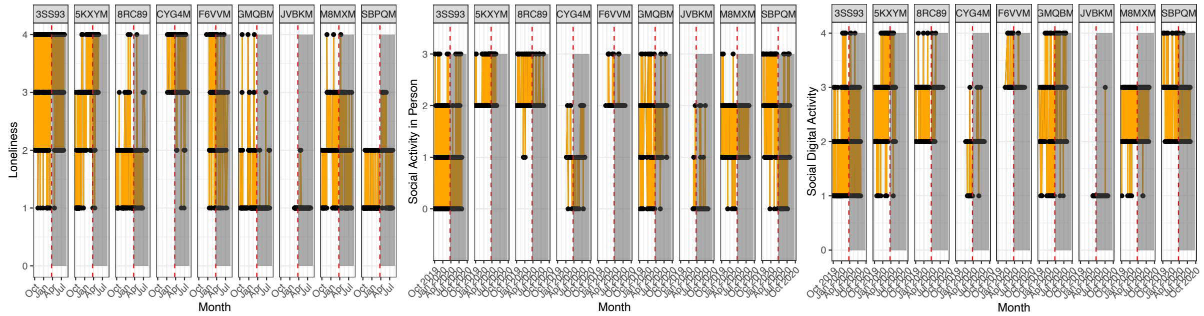

(A) (B) (C)

Figure 1S Survey data 2 months pre-post shelter-in-place orders (red line) for (A) loneliness, (B) social activity in person, (C) social digital activity.

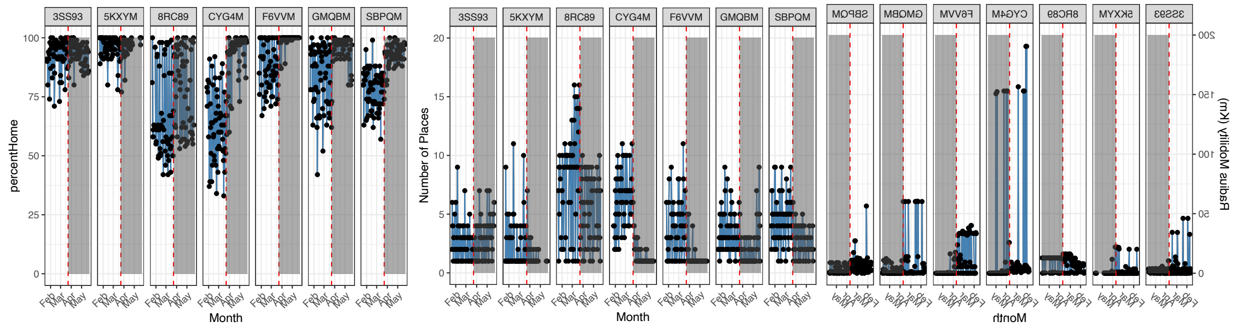

Figure 2S Survey data 2 months pre-post shelter-in-place orders (red line) for (A) percent of time spent at home, (B) number of places visited, (C) radius mobility in Km.

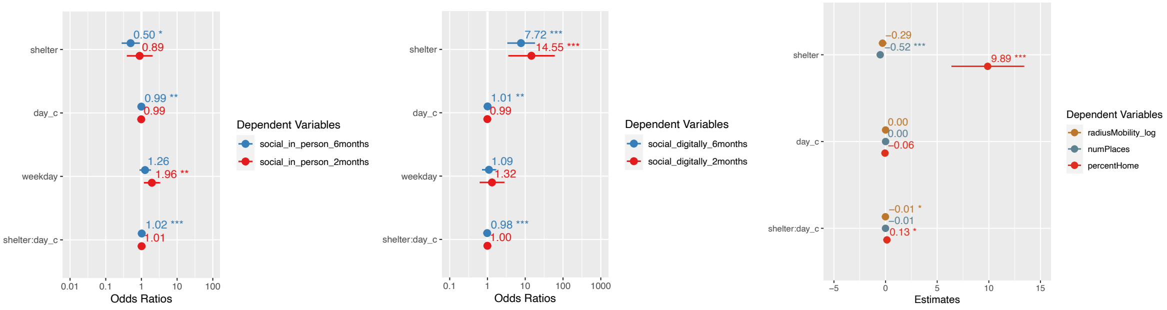

(A) (B) (C)

Figure 3S Generalized linear mixed model analyses for the effect of shelter-in-place orders on: (A) social activity in person including 2 months pre-post (red) including 6 months pre-post (blue); (B) social digital activity including 2 months pre-post (red) including 6 months pre-post (blue); (A) Radius mobility in Km (green), number of places visited (blue), percent of time spent at home (red) including 2 months pre-post shelter-in-place orders for a subset of 7 patients.
