# Supplementary material for: Intensive longitudinal assessment of mobility, social activity and loneliness in individuals with severe mental illness during COVID-19
Source: Schizophrenia (Heidelb). 2023 Sep 20;9(1):62. doi: 10.1038/s41537-023-00383-8 (PMC10511540; doi:10.1038/s41537-023-00383-8)
Supplement: Supplementary file 2 — Appendix [file 41537_2023_383_MOESM2_ESM.pdf]

Intensive Longitudinal Assessment of Mobility, Social Activity and Loneliness in Individuals with Severe Mental Illness During COVID-19

Authors: Linda Valeri, Habiballah Rahimi-Eichi, Einat Liebenthal, Scott Rauch, Russell Schutt, Dost Ongur, Lisa Dixon, *Jukka-Pekka Onnela, *Justin Baker

Co-last authors

eMethods Appendix

1. Overview of study design and study population

The Bipolar Longitudinal Study (BLS) is an ongoing longitudinal study that has thus far enrolled 74 patients with different psychotic and affective disorders since 2015 in Massachusetts. Participation required written informed consent. The study was approved by the local Institutional Review Board. Participants were asked to respond to a daily 31-item mobile survey questionnaire sent to them via the Beiwe smartphone application [1]. Patients participated in screening, baseline and monthly in-clinic or virtual Zoom visits throughout the follow-up that spanned from 1 week to 4 years. For this analysis, participants were included if they had at least two months of follow-up prior and post the shelter-in-place orders enacted on March 23, 2020 in Massachusetts. 9 participants met these criteria. Survey data were included up to six months prior and post the shelter in place orders. To maximize the quality of data processing, Global Positioning System (GPS), data available for 7 participants was included up to two months prior and post the shelter in place orders.

2. Smartphone data collection

Participants installed the Beiwe smartphone application [1] on their personal smartphones. Beiwe is an open-source, end-to-end encrypted digital phenotyping platform that consists of native Android and iOS smartphone applications, a web-based portal for study configuration, HIPAA-compliant cloud-based data storage processing back-end. The smartphone application was configured to collect location data using the GPS sensor for 90 seconds every 20 minutes to preserve the phone battery [2]. The application was also configured to send daily reminders to fill the survey. All data were collected and stored in compliance with local, state, and national laws, and all regulations and policies.

3. Study Measures

At baseline demographic information (age, gender, race) and patient's diagnosis were recorded. The daily 31-item survey included ordinal questions (scale: 0-4) on positive and negative mood, psychotic symptoms, alcohol and coffee consumption, social behavior, physical activity, and sleep. In our study, we considered the following survey items: social activity in person, digital social activity, and loneliness self-report. Primary predictor was the shelter-in-place order enacted on March 24, 2020, in Massachusetts. We furthermore processed GPS data using an open-source Deep Phenotyping of Location, DPLocate pipeline that first uses temporal filtering to detect Epochs. Aligned with the semi-continuous data collection setting; an Epoch was defined as a collection of 1 to 100 data points collected temporally adjacent to each other, and they were at

least 45 seconds apart from the neighboring Epochs. In case of detecting rare Epochs longer than 5 minutes, they were broken into smaller ones of less than 2 minutes long. Then, spatial clustering algorithm, which is a modified version of Density-Based Clustering Algorithm with Noise (DBSCAN), [3] was deployed to find the frequently visited Points of Interest (POIs) including Home, as the most visited place. The daily behavioral map of the individual as being present at the POIs was then used to obtain the mobility features: (1) Radius of mobility (Km): the radius of a circle that encompasses all Epochs visited in a day, (2) Home (%): percentage of the time the participant spends at home which could be the Home POI if visited on that day or otherwise, the most visited place of the day, (3) Places (#): number of POIs rather than Home that the individual has visited during the day.

4. Statistical Analysis

We provided descriptive statistics for relevant demographic factors (gender, age, diagnosis), for the survey measures of social activity, loneliness two and six months pre-and-post shelter in place orders and the passive mobility features two months pre-and-post shelter in place orders.

We used an interrupted time series approach to analyze before/after shelter-in-place orders behavior of patients (January 24, 2019 to March 24, 2020 for analyses including both GPS and survey data and September 24, 2019 to March 24 2020 for survey data only as secondary analysis) and after shelter-in-place orders (March 25 to May 24, 2020 for analyses including both GPS and survey data and March 25 to September 24, 2020 for survey data only as secondary analysis). We employed generalized linear mixed

models with logit link to evaluate the impact of the shelter in place orders on in-person and digital social activity along with loneliness perceptions. For the mobility measures, we evaluated the impact of the shelter in place orders on percent of daily time spent at home and logarithm of daily mobility radius (to reduce skewness of distribution) using a linear link and for the number of POIs using a Poisson link. In all models we included a fixed effect for time (in days), an indicator for whether a time was pre- or post- shelter-in-place orders, and an interaction between these two variables, a weekend indicator, and a random intercept for each participant. We specified an unstructured within-subject covariance. Further, we investigated whether the effect of shelter in place orders on loneliness was modified by social activity including social-activity and shelter in place orders indicator interaction.

We conducted longitudinal counterfactual simulation-based mediation analysis [4] to estimate the indirect effect of shelter-in-place orders on loneliness through the social activity and mobility factors that displayed a significant association with loneliness. The analysis was conducted using the R package mediation, which allows estimation of direct and indirect effects from mixed effect models[5]. Mediation analysis requires the adjustment for confounders of the mediator-outcome and exposure-outcome relationship[6]. The former no unmeasured confounding assumption is less likely to hold than the latter, as shelter-in-place orders can be considered as an exogenous event. Furthermore, mediation analysis requires correct specification of the mediator and outcome models.

All tests were two-tailed and a p-value threshold of 0.05 was used to evaluate significance. Statistical analysis was performed on Rstudio version 1.3.1073 [7]. We

conducted two sensitivity analyses. First, to assess robustness of causal inferences, in secondary analyses we adjusted all models for diagnosis; the study conclusions were unaffected, but some models did not converge. Second, we conducted time series analyses for two patients who were followed the year prior the COVID-19 pandemic to verify that no effects of the shelter-in-place order indicator are found in the prior year; our analyses showed that no effect of shelter-in-place order indicator was found in prior year.

5. References

[1] Torous J, Kiang MV, Lorme J, Onnela J. New tools for new research in psychiatry: a scalable and customizable platform to empower data driven smartphone research. JMIR Ment Health 2016 May 05;3(2):e16.

[2] Berry JD, Paganoni S, Carlson K, et al. Design and results of a smartphone-based digital phenotyping study to quantify ALS progression. Ann Clin Transl Neurol. 2019;6:873-881.

[3] X. Xu, J. Jäger, and H.-P. Kriegel, “A Fast Parallel Clustering Algorithm for Large Spatial Databases,” Data Mining and Knowledge Discovery, vol. 3, no. 3, pp. 263–290, 1999, doi: 10.1023/A:1009884809343.

[4] Imai, K., Keele, L., & Tingley, D. (2010). A general approach to causal mediation analysis. Psychological methods, 15(4), 309.

[5] Tingley, D., Yamamoto, T., Hirose, K., Keele, L., & Imai, K. (2014). mediation: R Package for Causal Mediation Analysis. Journal of Statistical Software, 59(5), 1–38. https://doi.org/10.18637/jss.v059.i05

[6] Valeri, L., & VanderWeele, T. J. (2013). Mediation analysis allowing for exposure–mediator interactions and causal interpretation: theoretical assumptions and implementation with SAS and SPSS macros. Psychological methods, 18(2), 137.

[7] RStudio Team (2020). RStudio: Integrated Development for R. RStudio, PBC, Boston, MA URL http://www.rstudio.com/.
